# Evaluation of Antibiotic Dissemination into the Environment and Untreated Animals, by Analysis of Oxytetracycline in Poultry Droppings and Litter

**DOI:** 10.3390/ani11030853

**Published:** 2021-03-17

**Authors:** Ekaterina Pokrant, Karina Yévenes, Lina Trincado, Gigliola Terraza, Nicolás Galarce, Aldo Maddaleno, Betty San Martín, Lisette Lapierre, Javiera Cornejo

**Affiliations:** 1 Department of Preventive Animal Medicine, Faculty of Veterinary and Animal Sciences, University of Chile, Santiago CP 8820808, Chile; kariyevenescoa@gmail.com (K.Y.); lina.trincado@ug.uchile.cl (L.T.); gigliola.terraza@ug.uchile.cl (G.T.); ngalarce@ug.uchile.cl (N.G.); llapierre@uchile.cl (L.L.); 2 Programa de Doctorado en Ciencias Silvoagropecuarias y Veterinarias, Campus Sur Universidad de Chile, Santa Rosa 11315, La Pintana, Santiago CP 8820808, Chile; amaddaleno@veterinaria.uchile.cl; 3 Laboratory of Veterinary Pharmacology (FARMAVET), Faculty of Veterinary and Animal Sciences, University of Chile, Santiago CP 8820808, Chile; bsmartin@uchile.cl

**Keywords:** oxytetracycline, 4-epi-Oxytetracycline, poultry droppings, poultry litter, broiler chicken, HPLC-MS/MS, antibiotic dissemination, sentinel birds

## Abstract

**Simple Summary:**

Oxytetracycline (OTC) is an antibiotic used mainly in feed and drinking water. OTC is poorly absorbed in the gastrointestinal tract of poultry; making droppings a potential route of dissemination of this antibiotic. The aim of this study was to evaluate the dissemination of oxytetracycline excreted from treated birds to the environment and other untreated animals (sentinels), through the analysis of their droppings and litter by HPLC-MS/MS following the end of treatment. In treated bird droppings, the average concentration of OTC+4-epi-OTC ranged from 347.63 to 2244.66 µg kg^−1^. OTC+4-epi-OTC in litter reached concentrations of 22,741.68 µg kg^−1^. Traces of OTC+4-epi-OTC were detected in the droppings and litter from sentinels. Therefore, OTC+4-epi-OTC can persist in the litter of treated animals at high concentrations and can be transferred to untreated birds that share the same environment. This exposure has the potential to increase the likelihood of selection of resistant bacteria in the environment.

**Abstract:**

Oxytetracycline (OTC) is widely used in broiler chickens. During and after treatment a fraction of OTC is excreted in its original form and as its epimer, 4-epi-OTC in droppings. To address the transfer of OTC into the environment, we evaluated the dissemination of OTC and 4-epi-OTC from treated birds to the environment and sentinels, through the simultaneous analysis of broiler droppings and litter. Male broiler chickens were bred in controlled conditions. One group was treated by orogastric tube with 80 mg kg^−1^ of OTC and two groups received no treatment (sentinels). OTC+4-epi-OTC were analyzed and detected by a HPLC-MS/MS post the end of treatment. The highest concentrations of OTC+4-epi-OTC were detected in the droppings of treated birds 14-days following the end of treatment (2244.66 µg kg^−1^), and one day following the end of treatment in the litter (22,741.68 µg kg^−1^). Traces of OTC+4-epi-OTC were detected in the sentinels’ droppings and litter (<12.2 µg kg^−1^). OTC+4-epi-OTC can be transferred from treated birds to the environment and to other untreated birds. The presence and persistence of OTC+4-epi-OTC in litter could contribute to the selection of resistant bacteria in the environment, increasing the potential hazard to public and animal health.

## 1. Introduction

Tetracyclines, which were discovered in the 1940s, are broad spectrum bacteriostatic antibiotics [1,2]. In some countries, such as Brazil and China these antibiotics can be used to promote growth in farm animals. However, this practice was banned in Europe in 2006 and in the USA in 2017 [3,4]. The third Annual Report of the World Organization for Animal Health (OIE) on the Use of Antimicrobial Agents in Animals, states that tetracyclines were the most used class of antimicrobial in 166 countries between 2015 and 2017 [5]. Currently, more than 20 tetracyclines are available; tetracycline, chlortetracycline, oxytetracycline, and doxycycline are the most used in veterinary medicine [3]. 

Oxytetracycline (OTC) is used in the poultry industry and is commonly administered to chickens through feed and drinking water to treat several diseases, such as chronic respiratory disease, infectious coryza, and fowl cholera [1,6,7]. When OTC is administrated to birds either for treatment or as growth promoter, residues of the antibiotic persist in their products and by-products, and are excreted in droppings [6,8,9,10,11]. Analyses of the OTC pharmacokinetics in broiler chickens suggests that OTC is poorly absorbed from the gastrointestinal tract of birds, and is therefore excreted in droppings at a higher concentration when administered orally [12,13]. Other studies have found antimicrobials in the feces of animals, such as swine and bovine. Oxytetracycline, doxycycline and sulfadiazine were most frequently detected, followed by tetracycline and other antimicrobials. Animals can excrete a significant proportion of the administered antibiotics (17–90%) unchanged or as active metabolites (epimers or isomers) of the parent antimicrobial [14]. 

Weakly acidic conditions favor the transformation of OTC to the 4-epi-oxytetracycline (4-epi-OTC) epimer, which has approximately 30% of the antibacterial activity of its precursor [15,16,17]. Wang et al. [16] showed that the peak concentration of 4-epi-OTC detected in the manure of treated pigs was 1337.08 mg kg^−1^ and that this metabolite influenced OTC degradation. Therefore, the European Union (EU) considers the sum of both OTC and 4-epi-OTC, as a marker to monitor OTC residues in animal products [18]. However, antibiotic residues are not monitored in non-edible by-products, such as chicken droppings which form an important part of broiler litter, along with feather remains, shavings and feed scraps [19].

Various studies have detected OTC in poultry droppings, manure, and litter. Zhao et al. [20] detected OTC in the manure of chickens, with a relatively high occurrence of 27.8% of sampled birds and a geometric mean of 1.55 mg kg^−1^. Similarly, Li et al. [21] confirmed the presence of OTC in dropping samples from 18 chicken farms in the northeastern provinces of China. Tetracyclines were the most prevalent of all antimicrobial families tested for in the study and the occurrence of OTC in chicken droppings was 44.4% of samples with a range of 0.54 to 4.57 mg kg^−1^ [21]. Further research in Iran has identified the concentration of OTC in broiler manure from 25 different farms, where the levels ranged from 0.047 to 13.77 mg kg^−1^ [22]. In this context, in Egypt, Mahmoud and Abdel-Mohsein [23], found OTC concentrations from 5.9–1.33 µg g^−1^ in poultry litter and droppings. 

In vitro analyses of OTC residues in manure suggest that the half-life is 8.1 days in 80% humidity [24]. Berendsen et al. [25] determined that OTC has a longer half-life than chlortetracycline (CTC) in stored broiler manure. The presence and persistence of OTC in droppings and poultry litter is relevant because the by-products are used in the feed of other production animals and are re-applied to land as fertilizer [14,26]. Also, poultry litter can contribute to the dissemination of antibiotics to the environment and other untreated animals through dust which can carry antibiotic residues [27]. The carryover of antibiotic residues to untreated animals through the environment has been experimentally demonstrated by Stahl et al. [28], where traces of sulfadiazine were detected in plasma and urine of untreated pigs kept in a barn that was previously used to treat pigs. The dissemination of OTC has not been fully elucidated and the presence and persistence of this antibiotic in droppings and litter represents a hazard to the environment, as well as to public and animal health. Therefore, it is important to determine the concentrations of OTC that persist in these animal waste.

Tetracyclines are used world-wide in animal production, increasing the likelihood for antimicrobial resistance. Thus, surveillance of these antibiotics is paramount [3]. Consequently, the aim of this study was to evaluate concentrations of OTC and 4-epi-OTC in the droppings and litter of therapeutically treated birds and also of untreated birds, kept in nearby pens, in order to assess antimicrobial environmental dissemination. For this purpose, an analytical methodology by high-performance liquid chromatography-tandem mass spectrometry (HPLC-MS/MS) was optimized and validated to ensure the reliability and precision of the results obtained in droppings and litter samples of treated and untreated animals.

## 2. Materials and Methods

### 2.1. Experimental Animals

Male broilers from the Ross 308 genetic line (Ross^®^, Aviagen Inc., Huntsville, AL, USA) were raised from birth in an experimental unit specially designed to carry out this study. In this experiment, 1.5 m^2^ corrals were conditioned with clean shavings that later became part of the birds’ litter. Environmental conditions such as temperature (25 ± 5 °C), humidity (50–60%) and ventilation were controlled. 

During the experiment, the birds had ad libitum access to water and non-medicated feed, which was previously analyzed by HPLC-MS/MS to verify the absence of OTC residues. The diets were formulated according to the nutritional requirements of the breed as recommended in the Aviagen™ manual [29]. 

The protocol for the management and monitoring of experimental birds was based on Law No. 20.380 “On the Protection of Animals” [30] and Directive 2010/63/EU on the protection of animals used for scientific purposes [31]. Regulation (EC) No 1099/2009 on the protection of animals at the time of killing [32] was respected. The Institutional Committee for the Comité Institucional de Cuidado y Uso de Animales (CICUA) of the University of Chile approved the use of the birds for the experimental study, certificate N°: 18187-VET-UCH-E1.

#### 2.1.1. Animal Groups and Treatment

**Figure 1:**
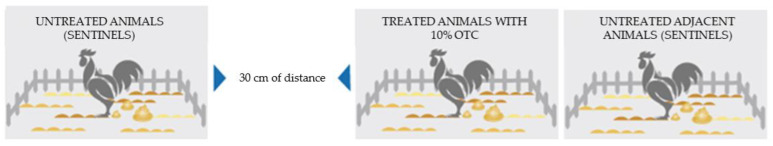
Schematic diagram of the placement of experimental groups; on the left group C, sentinels 30 cm from treated birds; in the center group A, birds treated with 80 mg kg^−1^ of OTC; and on the right group B, sentinels in adjacent pens.

In this study, the birds were randomly assigned from the first day of hatch to three experimental groups. Group A included 6 birds treated with a pharmaceutical formulation containing OTC at 100 mg mL^−1^ (10%), which is authorized for use in fattening birds. All birds in group A were treated orally using an orogastric tube (Levin No. 6) and a sterile syringe, with a therapeutic dose of 80 mg kg^−1^ for 10 consecutive days. The drug was administered directly into the crop, ensuring the delivery of calculated doses. The birds were weighed daily, and the dosage calculated according to individual weight was administered. (Tables S1 and S2). The second and third group (Groups B and C) also contained 6 birds each and were untreated (sentinel birds). To determine the transfer of OTC residues from treated birds to untreated birds group B was kept in an adjacent pen to group A while group C was kept 30 cm from group A (Figure 1). In this latter, an effective separation of 30 cm was determined in order to avoid the total direct contact between the birds. All pens had an area of 1.5 m^2^, divided by a solid wall of 1.5 m in height. All measures were taken to avoid contamination by handling, such as the use of shoe covers, gloves, and protective implements for the handling of each experimental group.

#### 2.1.2. Sampling Collection

**Figure 2:**
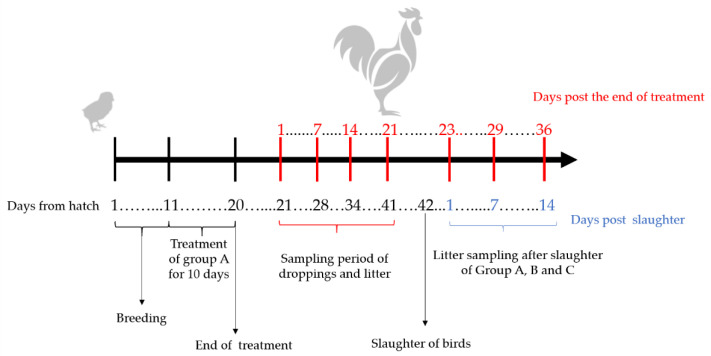
Schematic diagram of treatment and sampling period.

Six samples of droppings and litter were collected per sampling point, corresponding at days 1, 7, 14, and 21 post the end of treatment. Litter samples were also collected on days 1, 7, and 14 post-slaughter (up to 22 days post the end of treatment), to determine the persistence of OTC in the pens without experimental birds. The day of slaughter was at 42 days of hatch, corresponding to the slaughter day of broilers in the industry (Figure 2). 

Five grams of dropping were obtained from the cloaca of each bird by stimulation with sterile torulas and were stored individually in sterile polypropylene tubes at −20 °C until chemical analysis. Meanwhile, ten grams of litter samples were collected from Group A, B and C. Six samples were obtained at each time point in accordance with the Servicio Agrícola y Ganadero (SAG) soil sampling protocol, which establishes the requirements to comply with the parameters to be evaluated under Article 28 of the DS: No. 4/09 [33]. According to this. samples were collected equidistantly within each pen, covering the whole area, using an asystematic sampling method, as suggested by the protocol mentioned above. Litter samples were stored at −20 °C, in properly labelled plastic bags until further processing and analysis.

### 2.2. Chemical Analysis 

#### 2.2.1. Reagents, Solvents and Standards

For sample fortification, certified standards of OTC and 4-epi-OTC were purchased from Dr. Ehrenstorfer™. Tetracycline-d6 (TC-d6) was used as an internal standard (IS) and was purchased from Toronto Research Chemicals (Toronto, Canada). A stock solution of OTC, 4-epi-OTC and TC-d6 was prepared in methanol at a concentration of 1000 µg mL^−1^ and stored at −80 °C. Intermedia or working solutions were prepared using a diluted stock solution at a concentration of 1000 ng mL^−1^ and stored at −80 °C. 

Acetonitrile, methanol, and water were used for extraction of analytes from matrices. All solvents were from LiChrosolv^®^ (MERCK KGaA, Darmstadt, Germany) line and LC-MS grade. Buffer EDTA-McIlvaine was also used for the extraction procedure. It was prepared from 0.1 M citric acid and 0.2 M disodic phosphate. The McIlvaine buffer was prepared by mixing 500 mL of 0.1 M citric acid solution with 280 mL of 0.2 M disodium phosphate. The solution was brought to 2 liters with Milli-Q^®^ water and the pH adjusted to 4.0 ± 0.1 with 0.1 M citric acid or 0.2 M disodic phosphate. For the 0.1 M Na_2_EDTA-McIlvaine buffer, 74.4 g of Na_2_EDTA (Titriplex^®^ III ACS, ISO, Reag. Ph Eur (MERCK KGaA, Darmstadt, Germany) was dissolved with the EDTA-McIlvaine buffer previously prepared. 

For chromatography analysis, 0.1% formic acid in water with a pH 2.7 ± 0.2 (eluent A) and 0.1% formic acid in methanol with a pH 3.0 ± 0.3 (eluent B) were used. 

#### 2.2.2. Extraction Procedure

The extraction procedure was based on the previously published protocol of Berendsen et al. [13]. The protocol was optimized and validated for the detection of OTC and 4-epi-OTC in droppings and litter. For the analysis, 1 ± 0.01 g of sample was homogenized and weighed in a 50 mL polypropylene tube, 8 mL of EDTA-McIlvaine buffer solution and 2 mL of acetonitrile were used for extraction of OTC+4-epi-OTC. Samples were filtered through Whatman™ filter paper grade GF/A (1.6 µm) (MERCK) and then the extract was applied to a solid phase extraction column (SPE) Supel™ Select HLB (Supelco, MERCK KGaA, Darmstadt, Germany), previously conditioned with 5 mL of methanol and 5 mL of water LC-MS grade. The columns were then washed with 5 mL of water LC-MS grade, dried with a manifold pump for 5 min and finally eluted with 10 mL of methanol LC-MS grade. The samples were dried under a flow of mild nitrogen in a water bath between 40–50 °C in an automated solvent evaporation system (TurboVap^®^ LV, Biotage, Uppsala, Sweden) and reconstituted with 200 µL methanol and 300 µL water HPLC grade, and finally transferred to glass vials for chromatographic analysis by HPLC-MS/MS.

#### 2.2.3. Instrumental Analysis

All samples were analyzed using a high-performance liquid chromatography system, consisting of an Agilent 1290 Infinity autosampler and thermostat and Agilent 1260 Binary pump, coupled to a triple quadrupole tandem mass spectrometer, in multiple reaction monitoring mode (MRM) through an electrospray interface. An API 5500 (AB Sciex, Darmstadt, Germany) mass spectrometer was used. This device was operated in positive ionization mode for the detection of OTC, 4-epi-OTC, and TC-d6 analytes. 

The samples were processed and analyzed at the Laboratory of Veterinary Pharmacology (FARMAVET) of Faculty of Veterinary and Animal Sciences, of the University of Chile. This laboratory works under required biosecurity measures, good laboratory practices, and is accredited under ISO 17025 standards. 

### 2.3. Validation of Analytical Methodology 

Analytical methods are an essential tool for performing drug residue evaluation in edible and non-edible matrices. In order to ensure that the method is able to provide reliable and accurate data previous in-house laboratory validation is needed [34]. For this aim, validation of the method was performed through different procedures to ensure that the method detects and quantify OTC and 4-epi-OTC in droppings and litter by HPLC-MS-MS, precisely and confidently. 

The analytical method was validated following an internal protocol based on the recommendations from the regulation and guidance of Commission Decision 2002/657/EC [35] and VICH GL49 [36]. The parameters evaluated were retention time, limit of detection (LOD) and limit of quantification (LOQ), specificity, recovery, linearity of the calibration curve and precision (by repeatability and intra-laboratory reproducibility). LOD and LOQ of the analytical methodology, linearity of the calibration curve and method recovery, were considered as critical parameters for accurately quantify OTC residues in experimental samples. All parameters and acceptance criteria are described in Supplementary Table S4.

## 3. Results

### 3.1. Optimization and Validation of Analytical Method

During the implementation, the sample volume was decreased, and the volume of extraction solvents was increased. In addition, filtering the samples through Whatman™ microfiber glass paper filters, grade GF/A (1.6 µm) (MERCK) was added. These last two aspects were modified to improve the clean-up of the samples, since the droppings and poultry litter are highly complex matrices with many interferents that affect the reading and chromatographic analysis.

During the optimization, the optimal condition for chromatographic analysis were set. The reversed-phase column Sunfire ™ C18 3.5 µm, 2.1 mm × 150 mm (Waters Corp, Milford, MA, USA) was used for the analytical separation, with a temperature of 35 ± 1 °C, the injection volume corresponded to 20 µl, with a flow rate of 0.2 mL min^−1^. Eluent A was 0.1% formic acid in water with a pH 2.7 ± 0.2 and eluent B was 0.1% formic acid in methanol with a pH 3.0 ± 0.3. The analysis in the mass spectrometer was trough electrospray ionization (ESI), with a source temperature of 550 °C, and curtain and collision gas of 20 and 10 psi, respectively. The positive mode ion spray voltage was set at 45,000 V, and the ion source gas 1 and 2 at 60 and 80 psi, respectively. Table S3 shows the precursor and product ions for each analyte and the programming of the mass spectrometer to capture the respective ion products.

**Figure 3:**
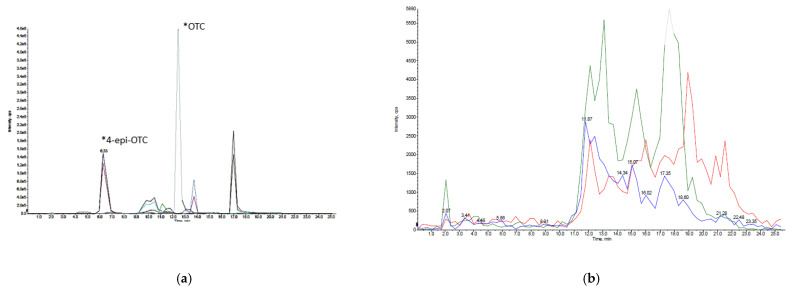
Representative chromatograms of the (**a**) injection of a certified standard of OTC and 4-epi-OTC (*: peaks) and from the analysis of (**b**) litter sample free of OTC and 4-epi-OTC.

All parameters, according to the internal validation protocol, fulfilled the criteria following guidelines 2002/657/EC and VICH GL49 [35,36] (Table S4). The average retention time for OTC (461.0/426.0 Da) was 11.632 ± 0.0571 min, and for 4-epi-OTC (461.0/426.0 Da) the average retention time was of 6.913 ± 0.0423 min (Figure 3). 

The method is specific for the detection of OTC and 4-epi-OTC, according to the analysis of 20 free samples, per study matrix. No interference in the retention times of OTC and 4-epi-OTC was detected in any replicates. In Figure 3, a chromatogram representative of the injection of a certified standard of OTC and 4-epi-OTC, and a litter sample free of these analytes is shown, in the latter no interference in the retention times is observed. 

The instrumental LOD and LOQ determined for OTC was 0.0027 and 0.0089 µg/g and 0.0023 and 0.0078 µg/g for 4-epi-OTC, respectively. The LOD and LOQ in droppings and broiler litter matrices are shown in Table 1.

The linearity for both analytes was determined by matrix curves fortified at concentrations of 12.5, 25, 50, and 100 µg kg^−1^ including zero, all curves showed a coefficient of determination (R^2^) greater than 0.99, in the two study matrices (Table 1). 

The relative standard deviation (RSD) determined for precision, through repeatability and reproducibility within the laboratory, did not exceed the 23% of variation, during the analysis of fortified samples at the lowest concentration of 25 µg kg^−1^ for both matrices. The results of the repeatability and reproducibility tests are described in the Supplementary Material (Table S5). For all levels of fortification, the recovery percentages were within the acceptable ranges of ±10% (Table 1 and Supplementary Table S5).

### 3.2. Detection and Quantification of OTC and 4-epi-OTC in Experimental Samples

Prior to the start of the study, bird droppings and shavings used for the bird litter were sampled. No OTC residues were detected in any of the samples. OTC and 4-epi-OTC concentrations were detected in the droppings of treated birds throughout the post the end of treatment period. It was observed that the highest concentrations of OTC+4-epi-OTC were quantified on day 14 post the end of treatment (2244.66 µg kg^−1^). OTC+4-epi-OTC concentrations were never below 300 µg kg^−1^ in treated birds (Table 2). Trace concentrations of OTC were detected groups B and C (sentinels). These levels were below the LOD in matrix (12.1 µg kg^−1^ for OTC and 12.2 µg kg^−1^ for 4-epi-OTC). An OTC+4-epi-OTC concentration of 9489.42 ug kg^−1^ was detected in only one dropping sample in Group C, 21 days after treatment (Table 2). For the analysis, the wet weight of the samples was considered. Droppings and litter samples had an average water content of 80% and 15%, respectively.

**Figure 4:**
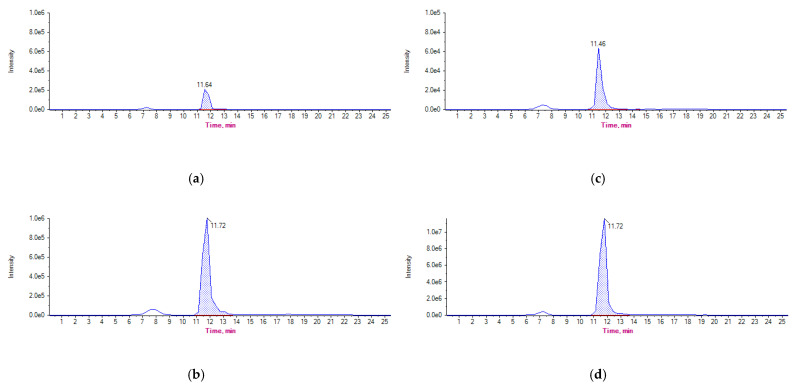
Chromatograms of OTC (461.0/261.0) from (**a**) droppings fortified at 50 µg kg^−1^, (**b**) experimental droppings sample from group A day 1 post the end of treatment, (**c**) litter sample fortified at 50 µg kg^−1^ and (**d**) experimental litter sample from group A day 1 post the end of treatment.

In group A, concentrations of OTC+4-epi-OTC in litter samples exceeded concentrations in droppings by more than 10 times one day following the end of treatment. A representative OTC (461.0/426.0) chromatogram of a dropping and litter sample at 50 µg kg^−1^ and OTC (461.0/426.0) chromatogram of experimental droppings and litter samples from the first sampling point of Group A are shown in Figure 4.

**Figure 5:**
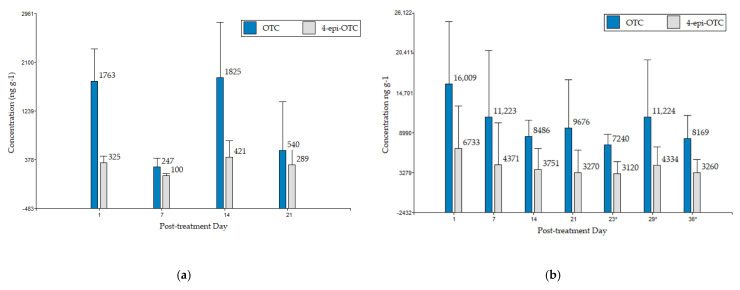
Concentrations of OTC and 4-epi-OTC in bird (**a**) droppings and (**b**) litter, from group A with 10% OTC, post the end of treatment and post slaughter (*: days post slaughter, correspond to 1-, 7- and 14-days post slaughter). Error bars represent the standard deviation.

In Figure 5, bar charts show the concentration of OTC and 4-epi-OTC separately, for group A. In litter OTC concentrations tend to decrease while the epimer is observed throughout. In droppings, concentrations varied throughout the course of sampling.

To determine differences between the concentration detected in droppings at days 1, 7, and 14 following the end of treatment, a *t*-test was performed. The detected concentrations were expressed as natural logarithm and the statistical software Infostat^®^ [37] was used for the analysis. In all experiments, differences were considered statistically significant when the associated probability level (*p*) was less than 0.05.

The analysis shows that for the concentrations of both analytes detected at seven days following the end of treatment, were statistically different than those detected on days 1 and 14 following the end of treatment (*p*-value < 0.05). While the concentrations detected for both analytes on days 1 and 14 do not present significant differences.

## 4. Discussion

The analytical procedure for OTC and 4-epi-OTC detection by HPLC-MS/MS was optimized from work previously published by Berendsen et al. [13] with the aim of extracting OTC and its epimer, from droppings and broiler litter. The modifications made were to improve the cleaning of the samples, in order to reduce the presence of interfering substances that could interfere with the chromatographic analysis, since as previously described, manure is a complex matrix [38]. The optimized analytical method can detect and quantify accurately and reliably, concentrations of OTC and 4-epi-OTC from dropping and litter matrices. All parameters determined during the validation of the methodology met the acceptance criteria according to the recommendations of 657/2002/EC and VICH GL49 [35,36]. The linearity of method presented a R^2^ greater than 0.99 for both sample types, although the dropping R^2^ was higher than broiler litter. The difference may be due to the fact that litter is a heterogeneous sample composed of different structures, such as feather and feed remains [19], which makes the processing of the sample during the extraction procedure more complex. The validated method is accurate and capable of determining the analytes of interest precisely over the concentration range encountered. In the analysis of experimental samples, high concentrations of OTC and its epimer were detected in treated bird droppings, which exceeded 2000 µg kg^−1^ on day 14 post the end of treatment. However, concentrations varied between samplings. This fluctuation in the droppings after the end of treatment may be due to the reabsorption or recirculation of OTC in the birds’ body to other organs or compartments. Odore et al. [39] detected OTC concentrations in treated broiler chicken bone tissue, this matrix is identified as a target tissue for tetracyclines, were a more complex link takes place between the tissue, calcium ions, and the rings of the basic tetracycline structure [39]. Moreover, this antibiotic is lipophilic with a large distribution volume, so high concentrations have been detected in different edible tissues, as liver, fat, kidney and muscle [40]. For this reason, and an increase in fat metabolism and recirculation from other tissues, the elimination of OTC could vary over time. Likewise, OTC and 4-epi-OTC quantified in droppings do not correlate with concentrations measured in edible tissues in previous studies, which evaluated the depletion of OTC and 4-epi-OTC residues in muscle, liver, claws and feathers samples from therapeutically treated birds [8,9]. The behavior of CTC and 4-epi-CTC, another tetracycline, has been studied in broiler droppings. CTC+4-epi-CTC residues of 179.45 µg kg^−1^ 25 days post the end of treatment were measured [41]. The samples showed microbial activity and the presence of resistance genes associated with tetracyclines [42]. Similarly, the concentrations of OTC+4-epi-OTC found in this study are of importance due to the likely selection of resistance in bacteria to these antimicrobials in the gastrointestinal tract of birds [43]. 

The quantified concentrations of OTC+4-epi-OTC in the litter of treated birds was 10 times greater than that found in droppings day 1 post the end of treatment. Increased concentrations of OTC in litter are likely due to the accumulation of bird droppings throughout the course of the experiment. Sarker et al. [44], analyzed litter samples for the detection of different antimicrobials. They determined that 23% of the samples were OTC positive, with an average concentration of 16.5 mg kg^−1^. Similar concentrations were observed in this study, whereby at day 7 following the end of treatment with OTC+4-epi-OTC the concentration in litter from the treatment group was 15.6 mg kg^−1^. We detected a decrease in OTC+4-epi-OTC over time. Then, 14 days post slaughter, the concentrations of these analytes were of 11.4 mg kg^−1^.

OTC+4epi-OTC persisted in the litter of treated birds post slaughter, at concentrations ranging from 10,360.60 to 15,557.62 µg kg^−1^. The high concentration of OTC+4-epi-OTC is the result of accumulation of excreted antibiotic during and post the end of treatment. Persistence of OTC+4-epi-OTC in the litter is due to the physical-chemical characteristics of the antibiotic. Kasumba et al. [45] investigated the anaerobic degradation of tetracyclines in the manure of different food-producing animal species and observed a 99-day half-life for OTC in poultry litter. They concluded that the persistence of OTC may be partly explained by the antibiotics ability to form stable complexes with divalent cations, as well as its capacity to adhere to proteins, particles, and organic matter [45]. 

Our results provide evidence suggesting that droppings may be a route of contamination and dissemination of OTC residues in the environment. Sentinel birds raised adjacent and at a distance of 30 cm from the treated birds, show trace concentrations of OTC in their litter and droppings. However, these concentrations could not be accurately quantified because the results obtained in the samples fluctuated between the LOD and LOQ of method. Detected trace concentrations show a chromatographic response of OTC greater than three times the signal noise of the instrumental baseline (Supplementary Figure S1). At 21 days post the end of treatment there was one positive litter sample in group C with a concentration of 9489.42 µg kg^−1^ of OTC+4-epi-OTC. It is possible that there was unintentional contamination to the area of the pen where the sample was taken during handling and/or feeding of the birds. In the same way, it could be attributed to the accumulation of OTC in the sampled area. 

Movement of the birds and the resulting dust in the air can result in the dissemination of OTC from treated to untreated birds [46]. The long half-life of OTC and the use of broiler droppings to fertilize soil also presents a risk for the transfer and persistence of OTC [47]. Oxytetracycline is known to bind to soil organic matter, clay minerals, and metal oxides [48]. OTC has been detected in soils in concentrations ranging from 0.3 to 300 mg kg^−1^ of soil [49]. 

Our results show that OTC is excreted from treated birds and can persists in litter, even up to 14 days post slaughter. In this study, a low probability of transfer was observed. This may be mainly due to the administration of the antimicrobial, since it was controlled through the nasogastric tube. Therefore, the factor of loss and dissemination of the antimicrobial through the drinking water or feed was not determined in this study. The carry-over and persistence of this antibiotic may be a risk for the development of antibiotic-resistant pathogens, due to selection pressure [50,51]. For this reason, it is necessary to monitor OTC residue transfer from animals and to investigate potential routes of contamination to help reduce the risk of antibiotic-resistance for both public and animal health. 

While the obtained results are interesting and raise some concerns that can be ad-dressed in further studies, some limitations should be recognized. The administration of the drug did not recreate industry conditions since the objective was to ensure the administration of the exact dose. Also, the number of replicates used was the minimum re-quired, in order to analyze more sampling points. Likewise, only one type on litter was study. Therefore, it would be interesting to take these aspects into account in future re-search in order to compare the results obtained and thus determine the factors that may play a role in the persistence and transfer of OTC residues into broiler litter. 

## 5. Conclusions

OTC and 4-epi-OTC in droppings and litter from therapeutically treated broiler chickens were detected following the end of treatment. High concentrations of OTC were excreted from treated birds, ranging from 347.63 µg kg^−1^ to 2244.66 µg kg^−1^ in their droppings. Concentrations of OTC+4-epi-OTC from the litter of treated birds ranged from 10,360.60 to 22,741.68 µg kg^−1^, and persisted 14 days post slaughter (11,429.14 µg kg^−1^). However, only trace concentrations of OTC were detected in droppings and litter from sentinel birds. These findings establish the first evidence that there is a low likelihood of the transfer of OTC residues from treated birds to the environment and to untreated birds in adjacent or separate pens, which needs to be further studied. 

## Figures and Tables

**Table 1:** Validation parameters for droppings and litter.

Matrix	Analyte	Linearity (R^2^) ^3^ ± SD	Recovery ^4^ (%)	LD ^5^ (µg kg^−1^)	LC ^6^ (µg kg^−1)^
Droppings	OTC ^1^	0.995 ± 0.004	104.3	12.128	36.384
	4-epi-OTC ^2^	0.997 ± 0.003	98.4	12.157	36.470
Litter	OTC	0.993 ± 0.004	91.9	12.409	37.228
	4-epi-OTC	0.992 ± 0.007	96.0	10.783	32.349

^1^ OTC: Oxytetracycline; ^2^ 4-epi-OTC: 4-epimer-Oxytetracycline; ^3^ Average R^2^ (Coefficient of determination) ± Standard deviation of 3 calibration curves in fortified matrix at a concentration of 12.5, 25, 50 and 100 µg kg^−1^, including zero; ^4^ Percentage of recovery from fortified matrix at a concentration of 25 µg kg^−1^; ^5^ Limit of Detection in matrix; ^6^ Limit of Quantification in matrix.

**Table 2:** OTC+4-epi-OTC concentrations in dropping and litter samples.

Days Post the End of Treatment (Days Post- Slaughter)	Average OTC+4-epi-OTC (µg kg^−1^) in Droppings			Average OTC+4-epi-OTC (µg kg^−1^) in Litter		
	Group A ^1^	Group B ^2^	Group C ^3^	Group A ^1^	Group B ^2^	Group C ^3^
1	2087.41	<LOD ^4^	<LOD *	22,741.68	<LOD	<LOD
7	347.63	<LOD	<LOD *	15,594.05	<LOD	<LOD
14	2244.66	<LOD	N/D ^5^	12,236.68	<LOD	<LOD
21	733.00	<LOD	N/D ^5^	12,946.13	<LOD	<LOD **
23 (1)	-	-	-	10,360.60	<LOD	<LOD
29 (7)	-	-	-	15,557.62	<LOD	<LOD
36 (14)	-	-	-	11,429.14	<LOD	<LOD

^1^ Treatment birds with 10% OTC; ^2^ untreated birds adjacent to treatment group; ^3^ untreated birds 30 cm from treated birds; ^4^ LOD: Limit of Detection (12.1 µg kg^−1^ for OTC and 12.2 µg kg^−1^ for 4-epi-OTC); ^5^ N/D: None Detected (no chromatographic signal of OTC and 4-epi-OTC above noise signal). * two samples show a response of OTC greater than 3 times the signal noise of the baseline. ** one sample measured 9489.42 µg kg^−1^ for OTC+4-epi-OTC.

## Data Availability

The data presented in this study are available in article.

## Supplementary Materials

The following are available online at https://www.mdpi.com/2076-2615/11/3/853/s1, Table S1. Weights of the experimental birds from group A, which were recorded during the treatment pe-riod, for calculation of the individual dose of oxytetracycline, Table S2. Individual dose of oxytetracycline administered to birds of group A birds for 10 consecutive days, Table S3. Substance specific mass spectrometric conditions, Table S4. Validation parameters and acceptance criteria following guidelines 2002/657/EC and VICH GL49 for validation of analytical methodology, Table S5. Validation of analytical methodology: Precision and recovery for droppings and litter, Figure S1: Representative OTC chromatograms of the first sampling (1-day post the end of treatment) from samples of (a) droppings from group B; (b) droppings from group C; (c) litter from group B; (d) litter from group C. The chromatographic signal of OTC reaches at least 3 times the signal noise of the baseline.

## References

[1] Chopra I., Roberts M. (2001). Tetracycline Antibiotics: Mode of Action, Applications, Molecular Biology, and Epidemiology of Bacterial Resistance. Microbiol. Mol. Biol. Rev..

[2] Daghrir R., Drogui P. (2013). Tetracycline Antibiotics in the Environment: A Review. Environ. Chem. Lett..

[3] Granados-Chinchilla F., Rodríguez C. (2017). Tetracyclines in Food and Feedingstuffs: From Regulation to Analytical Methods, Bacterial Resistance, and Environmental and Health Implications. J. Anal. Methods Chem..

[4] Roth N., Käsbohrer A., Mayrhofer S., Zitz U., Hofacre C., Domig K.J. (2019). The Application of Antibiotics in Broiler Production and the Resulting Antibiotic Resistance in Escherichia Coli: A Global Overview. Poult. Sci..

[5] World Organisation for Animal Health OIE Annual Report on Antimicrobial Agents Intended for Use in Animals 2018. https://www.oie.int/.

[6] Sumano López H., Gutiérrez Olvera L., Leon Fraga J., de León Fraga J. (2010). Farmacología Clínica en Aves Comerciales.

[7] Agyare C., Etsiapa Boamah V., Ngofi Zumbi C., Boateng Osei F., Kumar Y. (2019). Antibiotic Use in Poultry Production and Its Effects on Bacterial Resistance. Antimicrobial Resistance—A Global Threat.

[8] Cornejo J., Pokrant E., Krogh M., Briceño C., Hidalgo H., Maddaleno A., Araya-Jordán C., San Martín B. (2017). Determination of Oxytetracycline and 4-Epi-Oxytetracycline Residues in Feathers and Edible Tissues of Broiler Chickens Using Liquid Chromatography Coupled with Tandem Mass Spectrometry. J. Food Prot..

[9] Cornejo J., Pokrant E., Araya D., Briceño C., Hidalgo H., Maddaleno A., Araya-Jordán C., San Martin B. (2017). Residue Depletion of Oxytetracycline (OTC) and 4-Epi-Oxytetracycline (4-Epi-OTC) in Broiler Chicken’s Claws by Liquid Chromatography-Tandem Mass Spectrometry (LC-MS/MS). Food Addit. Contam. Part A.

[10] D’Angelo E. (2017). Sorption-Desorption Equilibrium and Diffusion of Tetracycline in Poultry Litter and Municipal Biosolids Soil Amendments. Chemosphere.

[11] Sarker Y., Hasan M., Paul T., Rashid S., Alam M., Sikder M. (2018). Screening of Antibiotic Residues in Chicken Meat in Bangladesh by Thin Layer Chromatography. J. Adv. Vet. Anim. Res..

[12] Ziółkowski H., Grabowski T., Jasiecka A., Zuśka-Prot M., Barski D., Jaroszewski J. (2016). Pharmacokinetics of Oxytetracycline in Broiler Chickens Following Different Routes of Administration. Vet. J..

[13] Berendsen B.J.A., Wegh R.S., Memelink J., Zuidema T., Stolker L.A.M. (2015). The Analysis of Animal Faeces as a Tool to Monitor Antibiotic Usage. Talanta.

[14] Massé D., Saady N., Gilbert Y. (2014). Potential of Biological Processes to Eliminate Antibiotics in Livestock Manure: An Overview. Animals.

[15] Lykkeberg A.K., Halling-Sørensen B., Cornett C., Tjørnelund J., Honoré Hansen S. (2004). Quantitative Analysis of Oxytetracycline and Its Impurities by LC-MS-MS. J. Pharm. Biomed. Anal..

[16] Wang Y., Chen G., Liang J., Zou Y., Wen X., Liao X., Wu Y. (2015). Comparison of Oxytetracycline Degradation Behavior in Pig Manure with Different Antibiotic Addition Methods. Environ. Sci. Pollut. Res..

[17] Li Z., Qi W., Feng Y., Liu Y., Ebrahim S., Long J. (2019). Degradation Mechanisms of Oxytetracycline in the Environment. J. Integr. Agric..

[18] (2010). EUROPEAN COMMISSION. Commission Regulation (EU) No 37/2010 of 22 December 2009 on Pharmacologically Active Substances and Their Classification Regarding Maximum Residue Limits in Foodstuffs of Animal Origin. Off. J. Eur. Union.

[19] Ghirardini A., Grillini V., Verlicchi P. (2020). A Review of the Occurrence of Selected Micropollutants and Microorganisms in Different Raw and Treated Manure—Environmental Risk Due to Antibiotics after Application to Soil. Sci. Total Environ..

[20] Zhao L., Dong Y.H., Wang H. (2010). Residues of Veterinary Antibiotics in Manures from Feedlot Livestock in Eight Provinces of China. Sci.Total Environ..

[21] Li Y., Zhang X., Li W., Lu X., Liu B., Wang J. (2013). The Residues and Environmental Risks of Multiple Veterinary Antibiotics in Animal Faeces. Environ. Monit. Assess..

[22] Alavi N., Babaei A.A., Shirmardi M., Naimabadi A., Goudarzi G. (2015). Assessment of Oxytetracycline and Tetracycline Antibiotics in Manure Samples in Different Cities of Khuzestan Province, Iran. Environ. Sci. Pollut. Res..

[23] Mahmoud M.A.M., Abdel-Mohsein H.S. (2019). Hysterical Tetracycline in Intensive Poultry Farms Accountable for Substantial Gene Resistance, Health and Ecological Risk in Egypt- Manure and Fish. Environ.l Pollu..

[24] Wang Q., Yates S.R. (2008). Laboratory Study of Oxytetracycline Degradation Kinetics in Animal Manure and Soil. J. Agric. Food Chem..

[25] Berendsen B.J.A., Lahr J., Nibbeling C., Jansen L.J.M., Bongers I.E.A., Wipfler E.L., van de Schans M.G.M. (2018). The Persistence of a Broad Range of Antibiotics during Calve, Pig and Broiler Manure Storage. Chemosphere.

[26] (2019). Kyakuwaire; Olupot; Amoding; Nkedi-Kizza; Basamba How Safe Is Chicken Litter for Land Application as an Organic Fertilizer? A Review. Int. J. Environ. Res. Public Health.

[27] Hartung J., Schulz J. (2008). Risks Caused by Bio-Aerosols in Poultry Houses. Poultry in the 21st Century: Avian Influenza and Beyond, 5–7 November 2007, Bangkok, Thailand.

[28] Stahl J., Zessel K., Schulz J., Finke J.H., Müller-Goymann C.C., Kietzmann M. (2016). The Effect of Miscellaneous Oral Dosage Forms on the Environmental Pollution of Sulfonamides in Pig Holdings. BMC Vet. Res..

[29] Aviagen Roos Nutrition Specifications 2019. https://tmea.aviagen.com/assets/Tech_Center/Ross_Broiler/RossBroilerNutritionSpecs2019-EN.pdf.

[30] Ministerio De Salud Subsecretaría De Salud Pública Ley Núm. 20.380 Sobre Protección de Animales 2009. https://www.bcn.cl/leychile/navegar?idNorma=1006858.

[31] Directive 2010/63/EU of the European Parliament and of the Council of 22 September 2010 on the Protection of Animals Used for Scientific PurposesText with EEA Relevance. 47. https://eur-lex.europa.eu/LexUriServ/LexUriServ.do?uri=OJ:L:2010:276:0033:0079:en:PDF.

[32] Council Regulation (EC) No 1099/2009 of 24 September 2009 on the Protection of Animals at the Time of KillingText with EEA Relevance. 30. https://eur-lex.europa.eu/LexUriServ/LexUriServ.do?uri=OJ:L:2009:303:0001:0030:EN:PDF.

[33] Servicio Agricola y Ganadero (SAG) Protocolo de Tomas de Muestras de Suelos 2019. https://www.sag.gob.cl/sites/default/files/Protocolo%20toma%20muestras%20suelo.pdf.

[34] European Medicines Agency (2006). ICH Topic Q 2 (R1) Validation of Analytical Procedures: Text and Methodology. https://www.ema.europa.eu/en/ich-q2-r1-validation-analytical-procedures-text-methodology.

[35] (2002). European Commission 2002/657/EC: Commission Decision of 12 August 2002 Implementing Council Directive 96/23/EC Concerning the Performance of Analytical Methods and the Interpretation of Results. Off. J. Eur. Union.

[36] European Medicines Agency (2015). VICH tpoic GL49: Studies to Evaluate the Metabolism and Residues Kinetics of Veterinary Drugs in Human Food-Producing Animals: Validation of Analytical Methods Used in Residue Depletion Studies. https://www.ema.europa.eu/en/vich-gl49-studies-evaluate-metabolism-residue-kinetics-veterinary-drugs-food-producing-animals.

[37] Grupo Infostat *Infostat Version 2020;* Facultad de Ciencias Agropecuarias, Universidad Nacional de Córdoba: Argentina. http://www.infostat.com.ar.

[38] Jansen L.J.M., van de Schans M.G.M., de Boer D., Bongers I.E.A., Schmitt H., Hoeksma P., Berendsen B.J.A. (2019). A New Extraction Procedure to Abate the Burden of Non-Extractable Antibiotic Residues in Manure. Chemosphere.

[39] Odore R., De Marco M., Gasco L., Rotolo L., Meucci V., Palatucci A.T., Rubino V., Ruggiero G., Canello S., Guidetti G. (2015). Cytotoxic Effects of Oxytetracycline Residues in the Bones of Broiler Chickens Following Therapeutic Oral Administration of a Water Formulation. Poult. Sci..

[40] Mestorino N., Hernández E.M., Marchetti L., Errecalde J.O. (2007). Pharmacokinetics and Tissue Residues of an Oxytetracycline/Diclofenac Combination in Cattle. Rev Sci Tech..

[41] Yévenes K., Pokrant E., Pérez F., Riquelme R., Avello C., Maddaleno A., Martín B.S., Cornejo J. (2018). Assessment of Three Antimicrobial Residue Concentrations in Broiler Chicken Droppings as a Potential Risk Factor for Public Health and Environment. Int. J. Environ. Res. Public Health.

[42] Cornejo J., Yevenes K., Avello C., Pokrant E., Maddaleno A., Martin B., Lapierre L. (2018). Determination of Chlortetracycline Residues, Antimicrobial Activity and Presence of Resistance Genes in Droppings of Experimentally Treated Broiler Chickens. Molecules.

[43] Manyi-Loh C., Mamphweli S., Meyer E., Okoh A. (2018). Antibiotic Use in Agriculture and Its Consequential Resistance in Environmental Sources: Potential Public Health Implications. Molecules.

[44] Sarker Y.A., Rashid Sm.Z., Sachi S., Ferdous J., Das Chowdhury B.L., Tarannum S.S., Sikder M.H. (2020). Exposure Pathways and Ecological Risk Assessment of Common Veterinary Antibiotics in the Environment through Poultry Litter in Bangladesh. J. Environ. Sci. Health B.

[45] Kasumba J., Appala K., Agga G.E., Loughrin J.H., Conte E.D. (2020). Anaerobic Digestion of Livestock and Poultry Manures Spiked with Tetracycline Antibiotics. J. Environ. Sci. Health B.

[46] Shields S.J., Garner J.P., Mench J.A. (2004). Dustbathing by Broiler Chickens: A Comparison of Preference for Four Different Substrates. Appl. Anim. Behav. Sci..

[47] Carballo M., Aguayo S., González M., Esperon F., Torre A. (2016). de la Environmental Assessment of Tetracycline’s Residues Detected in Pig Slurry and Poultry Manure. JEP.

[48] Jones A.D., Bruland G.L., Agrawal S.G., Vasudevan D. (2005). Factors Influencing the Sorption of Oxytetracycline to Soils. Environ. Toxicol. Chem..

[49] Cycoń M., Mrozik A., Piotrowska-Seget Z. (2019). Antibiotics in the Soil Environment—Degradation and Their Impact on Microbial Activity and Diversity. Front. Microbiol..

[50] Van Epps A., Blaney L. (2016). Antibiotic Residues in Animal Waste: Occurrence and Degradation in Conventional Agricultural Waste Management Practices. Curr. Pollut. Rep..

[51] Harada K., Asai T. (2010). Role of Antimicrobial Selective Pressure and Secondary Factors on Antimicrobial Resistance Prevalence in *Escherichia Coli* from Food-Producing Animals in Japan. J. Biomed. Biotechnol..

