# Supplementary material for: Evaluation of Antibiotic Dissemination into the Environment and Untreated Animals, by Analysis of Oxytetracycline in Poultry Droppings and Litter
Source: Animals (Basel). 2021 Mar 17;11(3):853. doi: 10.3390/ani11030853 (PMC8002629; doi:10.3390/ani11030853)
Supplement: Supplementary file 1 [file animals-11-00853-s001.pdf]

animals

MDPI

Supplementary Materials

Evaluation of antibiotic dissemination into the environment and untreated animals, by analysis of oxytetracycline in poultry droppings and litter.

Ekaterina Pokrant, Karina Yévenes, Lina Trincado, Gigliola Terraza, Nicolas Galarce, Aldo Maddaleno, Betty San Martín, Lisette Lapierre and Javiera Cornejo.

Table S1. Weights of the experimental birds from group A, which were recorded during the treatment period, for calculation of the individual dose of oxytetracycline.

Treatment day	Bird weight (g)					
	Bird 1	Bird 2	Bird 3	Bird 4	Bird 5	Bird 6
1	156	190	118	168	126	123
2	167	205	121	175	141	135
3	184	216	133	190	170	142
4	195	232	143	209	167	154
5	214	263	157	243	185	162
6	230	272	165	258	192	168
7	225	270	258	286	204	186
8	270	308	195	296	244	203
9	262	310	192	301	250	207
10	305	349	220	335	300	234

Table S2. Individual dose of oxytetracycline administered to birds of group A birds for 10 consecutive days.

Treatment day	Administered doses (mg)						Total[2]
	Bird 1	Bird 2	Bird 3	Bird 4	Bird 5	Bird 6	
1	12,48	15,2	9,44	13,44	10,08	9,84	70,48
2	13,36	16,4	9,68	14	11,28	10,8	75,52
3	14,72	17,28	10,64	15,2	13,6	11,36	82,8
4	15,6	18,56	11,44	16,72	13,36	12,32	88
5	17,12	21,04	12,56	19,44	14,8	12,96	97,92
6	18,4	21,76	13,2	20,64	15,36	13,44	102,8
7	18	21,6	12,64	22,88	16,32	14,88	106,32
8	21,6	24,64	15,6	23,68	19,52	16,24	121,28
9	20,96	24,8	15,36	24,08	20	16,56	121,76
10	24,4	27,92	17,6	26,8	24	18,72	139,44
Total[1]	176,64	209,2	128,16	196,88	158,32	137,12	1006.32

[1] Total oxytetracycline administered to each bird; [2] Total oxytetracycline administered per day of treatment.

Table S3. Substance specific mass spectrometric conditions.

Analyte	Precursor ion (m/z)	Product ion (m/z)	DP[4] (V)	EP[5] (V)	CE[6] (V)	CXP[7] (V)
OTC[1]	461.000	426.0[8]	72.0	10.0	28.0	25.0
		381.0	73.0		36.0	22.0
		444.0	70.0		30.0	15.0
4-epi-OTC[2]	461.000	426.0[8]	72.0	10.0	28.0	25.0
		381.0	73.0		36.0	22.0
		444.0	70.0		30.0	15.0
TC D6 (IS)[3]	451.000	160.0	34.0	10.0	25.0	30.0

[1] OTC: Oxytetracycline; [2] 4-epi-OTC: 4-epimer-oxyteracycline; [3] Tc D6 (IS): Tetracycline D6 (Internal Standard); [4] DP: Declustering potential; [5] EP: Entrance potential; [6] CE: Collision energy; [7] CXP: Collision cell exit potential; [8] quantitative ions.

Table S4. Validation parameters and acceptance criteria following guidelines 2002/657/EC and VICH GL49 for validation of analytical methodology.

Parameter	Description of analysis performed	Criteria
**Retention time**	6 injections of certified standard	RSD[1] < 2.5%
**LOD[2] and LOQ[3]**	1 calibration curve at concentrations of 0.005, 0.010, 0.020, 0.050 y 0.100 µg/mL (5, 10, 20, 50 y 100 µg kg^{-1}).	LOQ criteria: Signal-to-noise ratio 3:1 RSD ≤ 10%<br>LOD criteria: Signal-to-noise ratio 10:1 RSD ≤ 10%
**Linearity**	3 x 5 levels (0.25, 0.5, 1and 2 times the limit of 50 µg kg^{-1}, including zero)	R^2≥0.99<br>CV≤25%
**Recovery**	6 per level (one day) × 3 levels (0.5, 1, and 1.5 times the limit of 50 µg kg^{-1})	90%−110%
**Specificity**	20 blank samples	No interferences
**Repeatability (Precision)**	6 x 3 levels (0.5, 1, and 1.5 times the limit of 50 µg kg^{-1}), under the same conditions (one level per day)	RSD < reproducibility
**Reproducibility (Precision)**	6 x 3 levels (0.5, 1, and 1.5 times the limit of 50 µg kg^{-1}), under different conditions (one level per day)	RSD Between 16% and 23%
**LOD and LOQ in matrix**	7 repetitions at the level of the IQL[4]	LOQ criteria: Signal-to-noise ratio 3:1 RSD ≤ 10%<br>LOD criteria: Signal-to-noise ratio 10:1 RSD ≤ 10%

[1] Relative standard deviation; [2] Limit of detection; [3] Limit of quantification; [4] Instrumental quantification limit.

Table S5. Validation of analytical methodology: Precision and recovery for droppings and litter.

Analyte	Matrix	Work Concentration (μg kg^{-1})	RSD[3] of Repeatability (%)	RSD of Reproducibility (%)	Average Recovery (%)
OTC[1]	Droppings	25	3.33	4.74	104.3
		50	3.15	5.17	95.7
		75	1.09	1.63	101.4
	Litter	25	3.64	7.12	91.9
		50	3.54	6.06	108.1
		75	1.20	2.24	97.3
4-epi-OTC[2]	Droppings	25	3.32	21.30	98.4
		50	3.22	20.62	101.6
		75	1.10	7.02	99.5
	Litter	25	3.93	5.29	96.0
		50	3.82	4.89	104.0
		75	1.30	1.72	98.7

[1] OTC: Oxytetracycline; [2] 4-epi-OTC: 4-epimer-oxitetracycline; [3] Relative standard deviation.

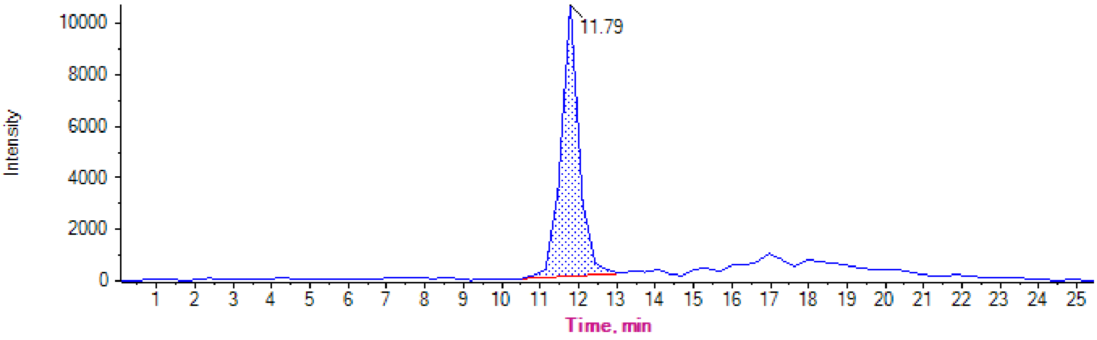

(**a**)

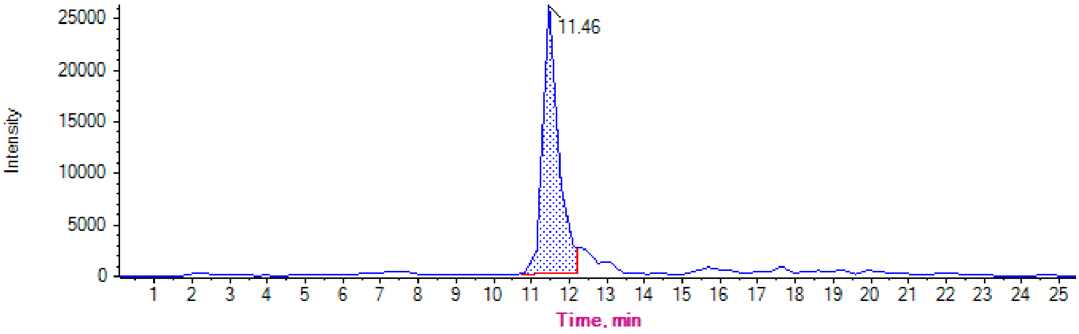

(b)

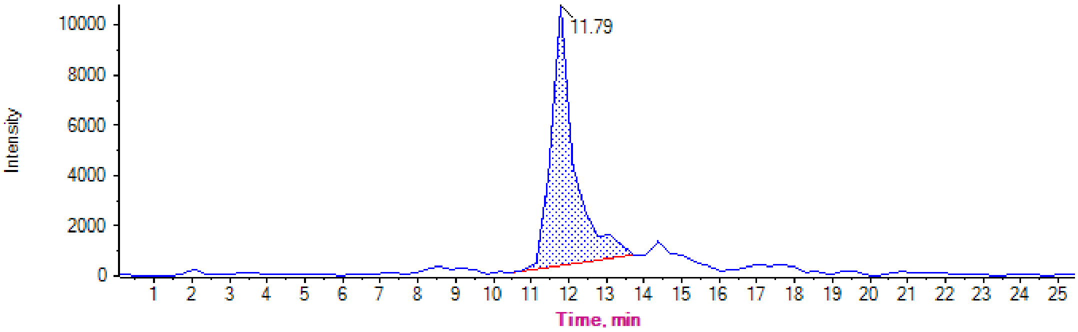

(c)

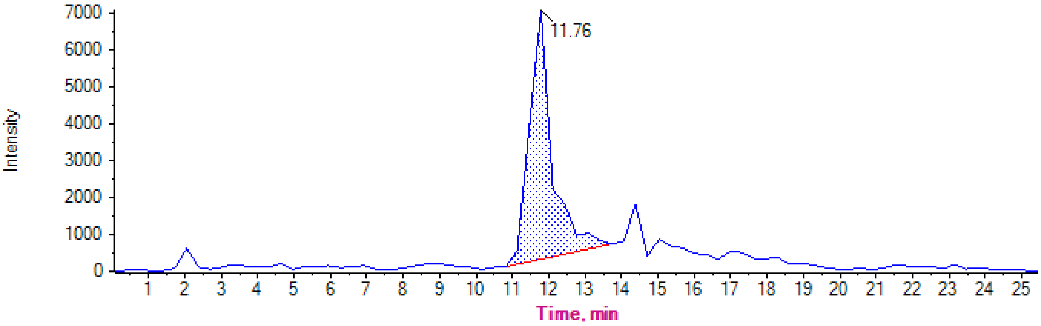

(d)

Figure S1. Representative OTC chromatograms of the first sampling (1-day post the end of treatment) from samples of (a) droppings from group B; (b) droppings from group C; (c) litter from group B; (d) litter from group C. The chromatographic signal of OTC reaches at least 3 times the signal noise of the baseline.
